# Annual acknowledgement of manuscript reviewers

**DOI:** 10.1186/1475-925X-13-13

**Published:** 2014-03-07

**Authors:** Kenneth R Foster

**Affiliations:** 1 Department of Bioengineering, University of Pennsylvania, Philadelphia, PA, USA

## Abstract

**Contributing reviewers:**

The Editor of BioMedical Engineering Online would like to thank all the Reviewers who have contributed to the journal in Volume 12 (2013).

## 

Vahid Abdollah

Hong Kong

Makoto Adachi

United States of America

Kerith Aginsky

South Africa

Bharath Ambale Venkatesh

United States of America

Kai-Nan An

United States of America

Maria Rosa Antognazza

Italy

Pasquale Aragona

Italy

Mina Arvin

Netherlands

Hiroshi Ashikaga

United States of America

Oleg Aslanidi

United Kingdom

René Attal

Austria

Ulas Bagci

United States of America

James Baish

United States of America

Alexandre Balbinot

Brazil

Rupak Banerjee

United States of America

Jayme Barbedo

Brazil

Brandon Bartling

United States of America

David Barton

United Kingdom

Berna Basarir

Turkey

Martin Bech

Germany

Balazs Benyo

Hungary

Enrique Berjano

Spain

Nacim Betrouni

France

Liam Blunt

United Kingdom

Frank Borg

Finland

Andrew Boryor

Germany

Marco Bottino

United States of America

Stuart Bowyer

United Kingdom

Stefan Braam

Netherlands

Andre Brown

United Kingdom

Christoph Brueser

Germany

Jonathan Brumberg

United States of America

Harvey Burd

United States Minor Outlying Islands

Wagner C A Pereira

Brazil

Giovanni Calcagnini

Italy

Luca Campana

Italy

Cristina M.R. Caridade

Portugal

Vincent Casey

Ireland

Joshua Cashaback

Canada

Francesco Cenni

Italy

Benedetta Cesqui

Italy

Hadrien Ceyte

France

Wallace Chamon

Brazil

Pak-Cheung Chan

Canada

Jin Ho Chang

Korea South

Geoff Chase

New Zealand

Mohd Zulfaezal Che Azemin

Malaysia

Guohua Chen

China

Houjin Chen

China

Jyh-Cheng Chen

Taiwan

Weng-Pin Chen

Taiwan

Wufan Chen

China

Yanxi Chen

China

Zhenglong Chen

China

Congo Tak-Shing Ching

Taiwan

Kyungeun Cho

Korea South

Young Cho

United States of America

Byoung Choi

Korea South

Kwok Wing Chow

Hong Kong

Blaine Christiansen

United States of America

Zhang Chunqiu

China

Edward Ciaccio

United States of America

Gastone Ciuti

Italy

Maureen Clerc

France

Ethan Cohen

United States of America

Damian Craiem

Argentina

Joel Cramer

United States of America

Saddha Cuijpers

Netherlands

Tien Tuan Dao

France

Joost de Jong

Netherlands

Junmin Deng

China

Thomas Desaive

Belgium

Thomas Desern

Germany

Giovanni Di Pino

Italy

Marco Di Rienzo

Italy

Alejandro Diaz-Sanchez

Mexico

Anh Dinh

Canada

Marcelo Do Amaral Ferreira

Brazil

Sebastian Doeltgen

Australia

Cyril Donnelly

Australia

Elisabetta Donzelli

Italy

Ivan Dotsinsky

Bulgaria

Thomas Doublet

France

Bin Du

China

Bin Duan

United States of America

John Dunlop

Germany

Matthieu Duvinage

Belgium

Andrzej Dziech

Poland

William Edwards

United States of America

Turgay Efe

Germany

Jan Egger

Germany

Anders Eklund

Sweden

Miroslawa El Fray

Poland

Irraivan Elamvazuthi

Malaysia

Ayman El-Baz

United States of America

Mohamed-Tarek El-Wakad

Egypt

Sauro Emerick Salomoni

Australia

Stephen Fairclough

United Kingdom

Yujiang Fan

China

Qingling Feng

China

Jinan Fiaidhi

Canada

Simonetta Filippi

Italy

Jorge L. Flores

Mexico

Brian Flynn

United Kingdom

Helene Follet

France

Hassan Fouad

Egypt

Massimiliano Fraldi

Italy

Piotr Franaszczuk

United States of America

Lorenzo Franchi

Italy

Yang-Chieh Fu

United States of America

Wei Gaofeng

China

Marco Antonio Garcia

Brazil

Marcos Garcia-Fuentes

Spain

Paolo Gargiulo

Iceland

Gunther Gerhardt

Brazil

Matthias Geyer

Germany

Vijay Goel

United States of America

Alex Golberg

Israel

Beat Göpfert

Switzerland

Elliot Greenblatt

United States of America

Jason Gu

Canada

Enrique Guerado

Spain

Berke Gur

Turkey

David Gutekunst

United States of America

Josef Guttmann

Germany

Taeyiung Ha

Korea South

Qing Hao

United States of America

Shiying Hao

United States of America

Abdelhamid Harabi

Algeria

Kamran Hassani

Iran

Jan Havlik

Czech Republic

Harun Taha Hayvaci

Turkey

Ralph Herkert

United States of America

Masaya Hirashima

Japan

Imo Hoefer

Netherlands

Markus Hoenicka

Germany

David Hoey

Ireland

Ales Holobar

Slovenia

Richard Hopkins

United States of America

Marc Horner

United States of America

Tim Howells

Sweden

Xiao Hu

United States of America

Hu Huang

United States of America

Changmo Hwang

Korea South

Ernesto Iadanza

Italy

Norliza Ibrahim

Malaysia

Nilesh Ingle

United States of America

David Int

Canada

Mikhail Inyushin

United States of America

Laura Lop

Italy

Muhammad Islam

Pakistan

Cinthia Itiki

Brazil

Marcus Jäger

Germany

In Gwun Jang

Korea South

Sabrina Yvonne Jauch

United Kingdom

Marc Jaumain

Belgium

Mervi Jehkonen

Finland

Matija Jezersek

Slovenia

Emil Jovanov

United States of America

Marianne Juhler

Denmark

Martti Juhola

Finland

Piotr Kaczmarek

Poland

Jacek Kadziela

Poland

Malik Kahook

United States of America

Maria Kallergi

Greece

Maarit Kangas

Finland

Dan Stieper Karbing

Denmark

Yuichi Kasai

Japan

Ulrich Katscher

Germany

Ira Katz

France

Ilda Kazani

Albania

Shawn Kelly

United States of America

Andy Kerr

United Kingdom

Donghyun Kim

Korea South

Hyunggun Kim

United States of America

Kyung Chun Kim

Korea South

Yeonhee Kim

United States of America

Youngho Kim

Korea South

Malte Kirst

Germany

Ching-Chang Ko

United States of America

Kranthi Kolli

United States of America

Grzegorz Konieczny

Poland

Robert Koprowski

Poland

Anna Korzynska

Poland

Yordan Kostov

United States of America

Boris Kotchoubey

Germany

Levente Kovacs

Hungary

Eddy Krueger

Brazil

Elizabeth Krupinski

United States of America

Przemyslaw Kryczka

Japan

Justin Laferrier

United States of America

Hsin-Yi Lai

Taiwan

Jonas Lantz

Sweden

Dmitry Laptev

Switzerland

Kirill Larin

United States of America

Martin Lawitzky

Germany

Soo Yeol Lee

Korea South

Chengwei Lei

United States of America

Xu Lei

China

Ney Lemke

Brazil

Haiyun Li

China

Huiqi Li

China

Junbo Li

China

Lieber Li

Taiwan

Yu-Shan Li

China

Zhi-Yong Li

China

Lun-De Liao

Singapore

Inger Lilliehöök

Sweden

Shang-Chih Lin

Taiwan

Madalena Lira

Portugal

Huafeng Liu

China

Jiang Liu

Singapore

Jie Liu

China

Wentai Liu

United States of America

Yi-Hung Liu

Taiwan

Yiyao Liu

China

Xianghong Luan

United States of America

Jianwen Luo

China

Jianhua Ma

China

Calixto Machado

Cuba

Ilia Makedonov

Canada

Jarmo Malinen

Finland

Denis Marcellin-Little

United States of America

Daniele Marinazzo

Belgium

Andrew Martin

United States of America

Daniel Martin

United Kingdom

Monika Martiniakova

Slovakia

Richard Mathias

United States of America

Eugenio Mattei

Italy

Dennis McFarland

United States of America

Paolo Melillo

Italy

Luciano Menegaldo

Brazil

Carlo Menon

Canada

Roberto Merletti

Italy

Lino Misoguti

Brazil

Christoph Moenninghoff

Germany

Iman Mohammad Rezazadeh

United States of America

Knut Möller

Germany

Juan C. Moreno

Spain

Roger Morrell

United Kingdom

Robert Motl

United States of America

Sayedali Mousavi

Iran

Michele Muccini

Italy

Joung H. Mun

Korea South

Marcelo Nacif

Brazil

Roozbeh Naemi

United Kingdom

Kweon-Ho Nam

Korea South

Valery Naranjo

Spain

Jonathan Newell

United States of America

Meir Nitzan

Israel

Tianye Niu

United States of America

Emil Novakov

France

Pascal Ntsama Eloundou

Cameroon

Riccardo Nucera

Italy

Shiro Oka

Japan

Felipe Orihuela-Espina

Mexico

Max Ortiz-Catalan

Sweden

Julien Oster

United Kingdom

Pertti Palo

United Kingdom

Libor Pasa

Czech Republic

Shamira Perera

Singapore

Angkoon Phinyomark

Thailand

Yang-Jun Pi

China

Kinga Pielichowska

Poland

Michael Plöchl

Germany

Christian Puttlitz

United States of America

Aike Qiao

China

Annalisa Quaini

United States of America

Timon Rabczuk

Germany

Paweł Ramos

Poland

Simon Rawlinson

United Kingdom

Sabrina Renzi

Italy

Maria Teresa Restivo

Portugal

Frances Robertson

South Africa

Jose F Rodriguez

Spain

Baxter Rogers

United States of America

Sebastien Roth

France

David Rubenstein

United States of America

Thorsten Rudroff

United States of America

Simo Saarakkala

Finland

Rosalind Sadleir

United States of America

Hassan Saeedi

Iran

Zahra Safaeepour

Iran

Jun Sakakibara

Japan

Solomon Samuel

United States of America

Saeid Sanei

United Kingdom

Bernd Saugel

Germany

Wolfram Schmidt

Germany

Gert Schoonenberg

Netherlands

Youngho Seo

United States of America

Toshihiro Sera

Japan

Hassan Serhan

United States of America

Igor Sersa

Slovenia

Suraj Shah

Canada

Jiawei

Shi China

Albert Shih

United States of America

Eun Bo Shim

Korea South

Minoru Shinohara

United States of America

Guofa Shou

United States of America

Sudhir Shrestha

United States of America

Emil Sidky

United States of America

Tobias Siebert

Germany

Gunter Siegmund

Canada

Fernando Silveira

Uruguay

Christer Sinderby

Canada

Sitabhra Sinha

India

Guanbin Song

China

Pavel Sovka

Czech Republic

Pascal Spincemaille

United States of America

Francesco Staffieri

Italy

Daniel Steines

United States of America

Berend Stoel

Netherlands

Abdulhamit Subasi

Bosnia and Herzegovina

Lei Sun

Hong Kong

Cathryn Sundback

United States of America

Tracey Swingler

United Kingdom

Eva Swinnen

Belgium

Hiroki Tamura

Japan

Xiaobo Tan

United States of America

Dalin Tang

United States of America

Joao Tavares

Portugal

Thomas Thuering

Switzerland

Luciana Torres

United States of America

Peppino Tropea

Italy

Khoa Truang Quang Dang

Vietnam

Vassiliy Tsytsarev

United States of America

Andrea Tura

Italy

Jan Turan

Slovakia

Giovanni Jacopo Ughi

Belgium

Costin Untaroiu

United States of America

Miguel Uva

Portugal

Liina Vahter

Estonia

Max Valentinuzzi

Argentina

Lex van Velsen

Netherlands

Emir Veledar

United States of America

Riccardo Vismara

Italy

Dung Vo

United States of America

Amy Wagoner Johnson

United States of America

Andrew Walker

Sweden

Eric Walker

United States of America

Baikun Wan

China

Mingxi Wan

China

Wan Mimi Diyana Wan Zaki

Malaysia

Gang Wang

China

Hongsheng Wang

United States of America

Shengzheng Wang

China

Shougang Wang

United States of America

Tao Wang

United States of America

Vicky Wang

New Zealand

Xintong Wang

United States of America

Yanzhen Wang

China

Beata Weglarz

Poland

Thomas White

United States of America

Oliver Wieben

United States of America

Slawomir Wilczynski

Poland

Ivo Wolf

Germany

Qingyu Wu

China

Shuicai Wu

China

Yunfeng Wu

China

Ming Xiao

United States of America

Hong-Bo Xie

Australia

Shane Xie

New Zealand

Lei Xing

United States of America

Xiao Yun Xu

United Kingdom

Xu Xu

China

Fraid Yaghouby

United States of America

Toshihiko Yamasaki

Japan

Cheng-Hong Yang

Taiwan

Chia-Yen Yang

Taiwan

Jikuang Yang

Sweden

Jinzhong Yang

United States of America

Xiaofeng Ye

China

Wei Yin

United States of America

Masaki Yoshida

Japan

Ningbo Yu

China

Rad Zdero

Canada

Zijing Zeng

United States of America

Weiqiang Zhang

China

Yuting Zhang

United States of America

Zhiqiang Zhang

United Kingdom

Gddr Zhao

China

Heng Zhao

United States of America

Guoyan Zheng

Switzerland

Jie Zheng

United States of America

Yu Zhou

United States of America

Peter Zilla

South Africa

Adam Zysk

United States of America

